# Dual functions of Rack1 in regulating Hedgehog pathway

**DOI:** 10.1038/s41418-020-0563-7

**Published:** 2020-05-28

**Authors:** Yan Li, Xiaohan Sun, Dongqing Gao, Yan Ding, Jinxiao Liu, Jiong Chen, Jun Luo, Junzheng Zhang, Qingxin Liu, Zizhang Zhou

**Affiliations:** 1 State Key Laboratory of Crop Biology, College of Life Sciences, Shandong Agricultural University, 271018 Tai’an, China; 2 State Key Laboratory of Pharmaceutical Biotechnology and MOE Key Laboratory of Model Animals for Disease Study, Model Animal Research Center, Nanjing University, 210061 Nanjing, China; 3 Department of Entomology and MOA Key Lab of Pest Monitoring and Green Management, College of Plant Protection, China Agricultural University, 100094 Beijing, China

**Keywords:** Proteolysis, Development

## Abstract

Hedgehog (Hh) pathway plays multiple roles in many physiological processes and its dysregulation leads to congenital disorders and cancers. Hh regulates the cellular localization of Smoothened (Smo) and the stability of Cubitus interruptus (Ci) to fine-tune the signal outputs. However, the underlying mechanisms are still unclear. Here, we show that the scaffold protein Rack1 plays dual roles in Hh signaling. In the absence of Hh, Rack1 promotes Ci and Cos2 to form a Ci–Rack1–Cos2 complex, culminating in Slimb-mediated Ci proteolysis. In the presence of Hh, Rack1 dissociates from Ci–Rack1–Cos2 complex and forms a trimeric complex with Smo and Usp8, leading to Smo deubiquitination and cell surface accumulation. Furthermore, we find the regulation of Rack1 on Hh pathway is conserved from *Drosophila* to mammalian cells. Our findings demonstrate that Rack1 plays dual roles during Hh signal transduction and provide Rack1 as a potential drug target for Hh-related diseases.

## Introduction

The evolutionarily conserved Hedgehog (Hh) pathway is first discovered in *Drosophila* through EMS-induced screening [1]. Following studies have demonstrated that Hh pathway is crucial for embryonic development and adult tissue homeostasis [2, 3], and its malfunction has been implicated in many human diseases [4, 5]. In the absence of Hh, the 12-transmembrane receptor Patched (Ptc) inhibits the cell surface localization of Smoothened (Smo), a 7-transmembrane GPCR-like transducer of Hh signaling [6]. Under this circumstance, the kinesin-related protein Costal2 (Cos2) recruits protein kinase A (PKA), casein kinase 1 (CK1), and glycogen synthase kinase 3 (GSK3) to the unique transcription factor Cubitus interruptus (Ci) to promote Ci phosphorylation [7]. In turn, the phosphorylated Ci protein is recognized and ubiquitinated by Slimb-Cul1 E3 ligase to partially degrade into a truncated form of Ci (Ci-75), which enters into the nucleus and acts as a repressor to inhibit the expression of target genes [8–10]. In the presence of Hh, secreted Hh proteins bind Ptc and co-receptors to relieve the inhibitory effect of Ptc on Smo, which leads to Smo cell membrane accumulation and Hh pathway activation [11, 12]. Hh signaling dissociates Ci–Cos2–kinases complexes and thereby blocks Ci phosphorylation and partial degradation, resulting in full-length Ci translocation into the nucleus to turn on the expression of target genes, such as *decapentaplegic* (*dpp*), *ptc*, and *rdx* [13, 14]. As a negative feedback, Rdx forms an E3 ligase complex with Cul3 to ubiquitinate Ci in the nucleus, leading to Ci complete destabilization, and Hh signaling termination [14, 15]. Contradictorily, Hh also hampers Rdx-mediated Ci proteolysis through promoting Ci interaction with its deubiquitinase Usp7 [16], suggesting that Hh pathway possibly plays distinct roles in regulating Ci abundance. To date, the mechanisms by which Hh dissociates Ci–Cos2–kinases association and promotes Smo cell surface expression are still unclear. The previous studies have showed that Hh stimulates Cos2 differential phosphorylation on S572 and S931 [17], but the phosphorylation is not required for normal activation of Ci by Hh [18], suggesting that other mechanism is involved in this process.

Another key component of Hh pathway is Smo, a GPCR-like transmembrane protein. Its cell surface accumulation is an essential step for Hh pathway activation. However, the underlying mechanisms governing Smo localization are still elusive. Inactivation of ubiquitin-activating enzyme Uba1 leads to Smo cell surface accumulation in *Drosophila* wing discs regardless of whether Hh is present [19], indicating that the subcellular localization of Smo is tightly controlled by ubiquitin modification. Consistently, several E3 ligases accounting for Smo ubiquitination have been identified recently, including HERC4, Smurf and Cul4-DDB1 [20–22]. The ubiquitination of Smo is a reversible process due to the function of deubiquitinating enzymes Usp8 and UCHL5 [19, 23, 24]. Although Hh signaling could promote Smo cell membrane expression through enhancing Smo association with the deubiquitinases [24], the detail mechanism how Hh promotes Smo binding to its deubiquitinases is still unclear.

The protein Rack1 (receptor for activated C-kinase 1) was originally identified as a receptor for protein kinase C [25]. It interacts with several C-kinase family members to activate their kinase activity [26]. In contrast, Rack1 binds Src kinase to inhibit its kinase activity [27, 28], indicating Rack1 shows activator or suppressor role for distinct kinases. Besides, it is well established that Rack1 associates with many proteins through its tandem WD40 domains. As a scaffold protein, Rack1 can recruit two or more molecules together to form a complex that has an important role in various cellular processes [26]. For example, Rack1 binds with c-Jun and ubiquitin ligase Fbw7 to form a complex, leading to c-Jun degradation and JNK pathway inactivation [29]. Rack1 also regulates autophagy via facilitating the assembly of Atg14L–Beclin1–Vps34–Vps15 complex [30]. Besides, Rack1 suppresses canonical Wnt signaling through stabilizing the β-Catenin destruction complex [31]. Therefore, Rack1 possibly plays multifaceted roles in a variety of physiological and pathological processes.

To find novel components of Hh signaling, we carried out a genetic screen and identified Rack1 as a regulator of Hh pathway. Knockdown of *rack1* increased Ci and Hh target genes expression, while overexpression of *rack1* showed opposite results. Through genetic and biochemical analyses, we demonstrated that Rack1 interacted with both Ci and Cos2 and enhanced Ci–Cos2 association, resulting in Slimb-mediated Ci proteolysis. Intriguingly, with Hh stimulation, Rack1 dissociated from Ci–Cos2 complex and formed a trimeric complex with Smo and Usp8, leading to Smo deubiquitination and cell surface accumulation. These results revealed that Rack1 plays dual roles in coordinating Hh pathway activity. Furthermore, the mammalian ortholog of Rack1 had a similar role in regulating Hh pathway. Taken together, our study demonstrated that the Rack1 plays dual roles in Hh pathway, and provided Rack1 as a prudent drug target for Hh-related cancers.

## Results

### Loss of *rack1* increases Ci protein level and Hh pathway activity

In the *Drosophila* wing, *hh* expresses exclusively in the posterior (P) compartment cells, while the unique transcription factor *ci* only expresses in the anterior (A) cells. After autoproteolytic cleavage and modification, the mature Hh protein is secreted from the P cells and moves to A cells to form a concentration gradient [32]. Thus, the Hh pathway is only activated in the A cells adjacent to the A/P boundary [2]. It has been documented that the activity of Hh pathway determines the intervein region between L3 and L4 of adult wing [33]. Upregulation of Hh signaling enhances the area of L3/L4 intervein, and vice versa. To identify novel Hh pathway regulators, we performed a RNAi-based genetic screening, in which individual RNAi lines were expressed in wing discs via *nub*-gal4 driver. Through this screening, we found that knockdown of *rack1* apparently increased the area of L3/L4 intervein (Supplementary Fig. S1a, b). In addition, knockdown of *rack1* resulted in the loss of cross vein between L3 and L4 (Supplementary Fig. S1a, b), indicating Hh pathway is hyperactivation.

**Fig. 1:**
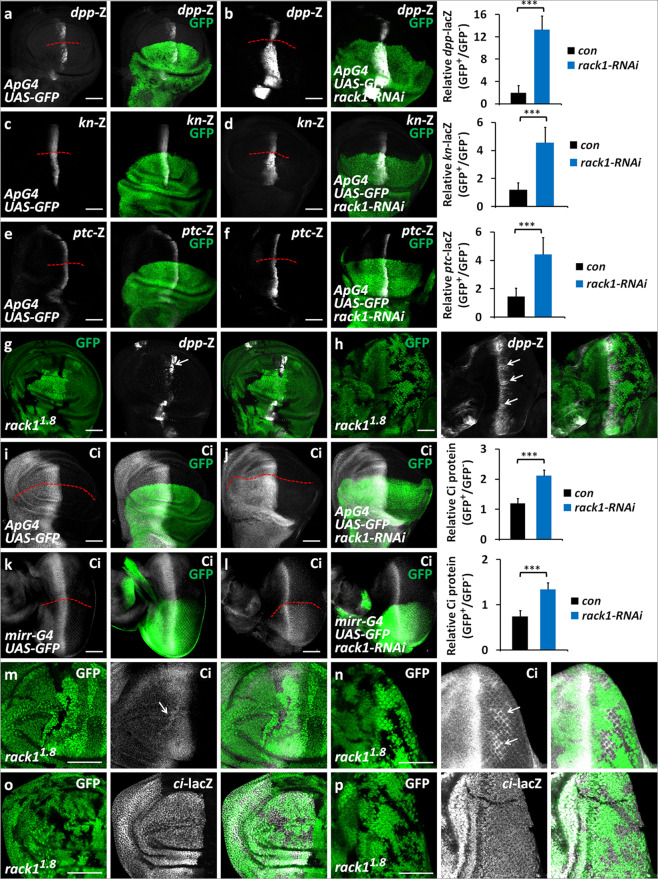
Loss of *rack1* activates Hh signaling and increases Ci protein level. All wing imaginal discs shown in this study were oriented with anterior on the left and ventral on the top. **a**, **b** Wing discs of control (**a**) or expressing *rack1* RNA interference (RNAi) by *ApG4* (**b**) were stained to show GFP (green) and *dpp*-lacZ (white). GFP marks the *apG4*-mediated gene expression pattern. *ApG4* drives *UAS* transgenes to be specifically expressed in the dorsal region of wing discs. Quantification analyses were shown on right (*n* = 5). **c**–**f** Wing discs of control (**c**, **e**) or expressing *rack1* RNAi (**d**, **f**) were stained to show GFP (green) and *kn*-lacZ (white in **c**, **d**), or *ptc*-lacZ (white in **e**, **f**). Quantification analyses were shown on right (*n* = 5). **g**, **h** A wing disc (**g**) or eye disc (**h**) carrying *rack1*^*1.8*^ mutant clones was immunostained to show the expression of GFP (green) and *dpp*-lacZ (white). Mutant clones are recognized by the lack of GFP. Of note, *rack1* mutant cells exhibited increase of *dpp*-lacZ (arrows). **i**, **j** Wing discs of control (**i**) or expressing *rack1* RNAi via *ApG4* (**j**) were stained to show GFP (green) and Ci (white). Quantification analyses were shown on right (*n* = 5). **k**, **l** Eye discs of control (**k**) or expressing *rack1* RNAi by *mirr-G4* (**l**) were stained to show GFP (green) and Ci (white). Quantification analyses were shown on right. **m**–**p** Wing discs (**m**, **o**) or eye discs (**n**, **p**) carrying *rack1*^*1.8*^ clones were stained to show GFP (green) and Ci (white in **m**, **n**), or *ci*-lacZ (white in **o**, **p**). Of note, loss of *rack1* mutant increased of Ci protein (marked by arrows in **m**, **n**), but did not affect *ci*-lacZ levels (**o**, **p**). Scale bars: 50 μm for all discs.

To validate whether Rack1 is involved in Hh pathway regulation, we examined the transcription of Hh pathway targets using lacZ reporters. Compared with the control disc (Fig. 1a), expression of *rack1* RNAi (#104470, VDRC) by *ApG4* apparently upregulated *dpp*-lacZ level (Fig. 1b). In addition, knockdown of *rack1* also increased *kn*-lacZ and *ptc*-lacZ expression (Fig. 1c–f), suggesting that *rack1* knockdown promotes the expression of both low- and high-threshold Hh target genes. On the other hand, we employed RT-PCR assay to validate that *rack1* RNAi decreased the mRNA levels of *ptc*, *dpp* and *kn* in wing discs (Supplementary Fig. S1c). To remove the off-target effect of *rack1* RNAi, we used a null allele of *rack1*, *rack1*^*1.8*^ (#24152, BDSC), which the glutamine on 6 is replaced by a stop codon (Q6stop). Since *rack1*^*1.8*^ fly was homozygous lethal at first instar larval stage, we employed FLP/FRT-mediated mitotic recombination and examined the expression of Hh target genes [34]. *rack1*^*1.8*^ homozygous clones, which were marked by loss of GFP expression, showed increased Ptc protein level (Supplementary Fig. S2a). In both wing (Fig. 1g) and eye discs (Fig. 1h), the expression of *dpp*-lacZ was upregulated in *rack1*^*1.8*^ mutant cells, indicating that loss of *rack1* indeed activates Hh pathway.

Since loss of *rack1* promotes the expression of several Hh target genes, including *dpp*, *kn*, and *ptc*, we speculated that Rack1 possibly regulates a key component of this pathway. We examined Ci protein levels using a rat anti-Ci antibody (2A1, DSHB), which only recognizes the full-length Ci [35]. Compared with controls (Fig. 1i, k), knockdown of *rack1* upregulated Ci protein levels in both wing and eye discs (Fig. 1j, l). We confirmed this result using another RNAi line (#34694, BDSC), which targets distinct region of *rack1* (Supplementary Fig. S2b, c). Furthermore, loss of *rack1* upregulated the full-length Ci (Fig. 1m, n), but did not show any detectable effect on *ci* transcription as monitored by the expression of *ci*-lacZ (Fig. 1o, p), an enhancer trap that mimics *ci* transcription, indicating that Rack1 possibly regulates Ci through controlling Ci protein. Taken together, these results show that Rack1 is a bona fide regulator of Hh signaling.

### Rack1 is involved in Slimb-mediated Ci degradation

Since the previous studies have demonstrated that Rack1 enhances PKC kinase activity [25], while suppresses Src activity [36], we next sought to test whether Rack1 regulates Hh pathway through PKC or Src kinase. The *Drosophila* genome encodes five PKC homologs (PKC53E, PKC98E, PKN, PKCδ, and aPKC) and two Src homologs (Src42A and Src64B). Compared with the control disc (Supplementary Fig. S3a), knockdown of *rack1* increased *ptc*-lacZ expression in the anterior compartment (Supplementary Fig. S3b), whereas knockdown of any *PKCs* did not affect *ptc*-lacZ level (Supplementary Fig. S3c–g). In addition, overexpression of a highly specific pseudosubstrate PKCi was able to inhibit all PKC activities [37], but did not change *ptc*-lacZ expression (Supplementary Fig. S3h), removing the possibility that Rack1 modulates Hh pathway via PKCs. On the other hand, knockdown of *Src42A* or *Src64B* did not affect *ptc*-lacZ level (Supplementary Fig. S3i, j).

**Fig. 2:**
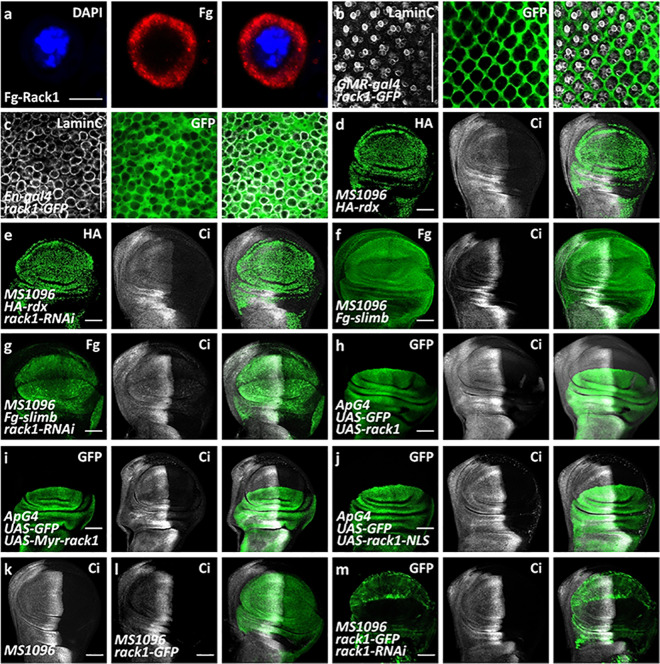
Rack1 regulates Slimb-mediated Ci proteolysis. **a** A S2 cell transfected with Fg-Rack1 was stained by DAPI (blue) and Fg (white). Fg-Rack1 mainly localized in the cytoplasm. **b**, **c** A eye disc (**b**) or wing disc (**c**) expressing *rack1*-GFP was stained with Lamin C (white) and GFP (green). **d**, **e** Wing discs expressing HA-*rdx* only (**d**), or HA-*rdx* plus *rack1* RNAi simultaneously (**e**) were stained to show HA (green), and Ci (white). **f**, **g** Wing discs expressing Fg-*slimb* only (**f**), or Fg-*slimb* plus *rack1* RNAi simultaneously (**g**) were stained to show Fg (green) and Ci (white). **h**–**j** Wing discs expressing *rack1* (**h**), Myr-*rack1* (**i**) or *rack1*-NLS (**j**) were stained to show GFP (green) and Ci (white). Notably, Rack1 and Myr-Rack1 decreased Ci, but Rack1-NLS did not show any effect on Ci. **k**–**m** Wing discs of control (**k**), expressing *rack1*-GFP (**l**), or expressing *rack1*-GFP plus *rack1* RNAi simultaneously (**m**) were stained to show GFP (green) and Ci (white). Overexpression of *rack1*-GFP could counteract *rack1* RNAi-mediated Ci increase. Scale bars: 2 μm for S2 cell (**a**), while 50 μm for all disc (**b**–**m**).

As a matter of fact, Ci protein is subjected to degradation by dual pathways [38]. In the cytoplasm, Slimb-Cul1 promotes Ci partial degradation to generate a truncated form Ci-75, while in the nucleus, Ci protein is destabilized by Rdx-Cul3 E3 ligase [38]. Thus, Ci protein undergoes distinct degradation in cytoplasm and nucleus. Through immunostaining, we found that the Rack1 protein predominantly localized in the cytoplasm in S2 cells (Fig. 2a), eye disc cells (Fig. 2b), and wing disc cells (Fig. 2c), indicating that Rack1 likely affects cytoplasmic Ci. Consistently, knockdown of *rack1* did not restore Rdx-induced Ci decrease (Fig. 2d, e), but effectively neutralized Slimb-mediated Ci proteolysis in the anterior compartment cells (Fig. 2f, g).

Because our above data showed that loss of *rack1* increased Ci protein, we next examined whether ectopic expression of *rack1* decreased Ci. Compared with the control disc (Fig. 1i), overexpression of *rack1* indeed resulted in Ci decrease (Fig. 2h). On the other hand, ectopic expression of *rack1*-Y229/247F, a mutant form fails to bind Src kinase, also decreased Ci (Supplementary Fig. S3k), demonstrating that Rack1 regulates Hh pathway in a Src-independent manner. We also generated a membrane-tethered form of Rack1 (Myr-*rack1*) by adding a myristoylation signal at its N-terminus. Overexpression of Myr-*rack1* decreased Ci protein (Fig. 2i), whereas the nuclear localized Rack1 (*rack1*-NLS, a nuclear localization sequence fused to its C-terminus) failed to affect Ci (Fig. 2j), supporting that only cytoplasmic Rack1 is involved in Ci regulation. Consistently, overexpression of *rack1* did not decrease Ci under *slimb*-RNAi background (Supplementary Fig. S4a–f), suggesting that Rack1 downregulates Ci depending on Slimb. In addition, we showed that overexpression of *rack1* could restore *rack1* RNAi-induced Ci decrease (Fig. 2k–m), further excluding the off-target effect of *rack1* RNAi. Collectively, these data suggest that Rack1 regulates Hh pathway possibly through affecting Slimb-Cul1-mediated Ci proteolysis in the cytoplasm.

### Rack1 synergizes with Cos2 to decrease Ci

**Fig. 3:**
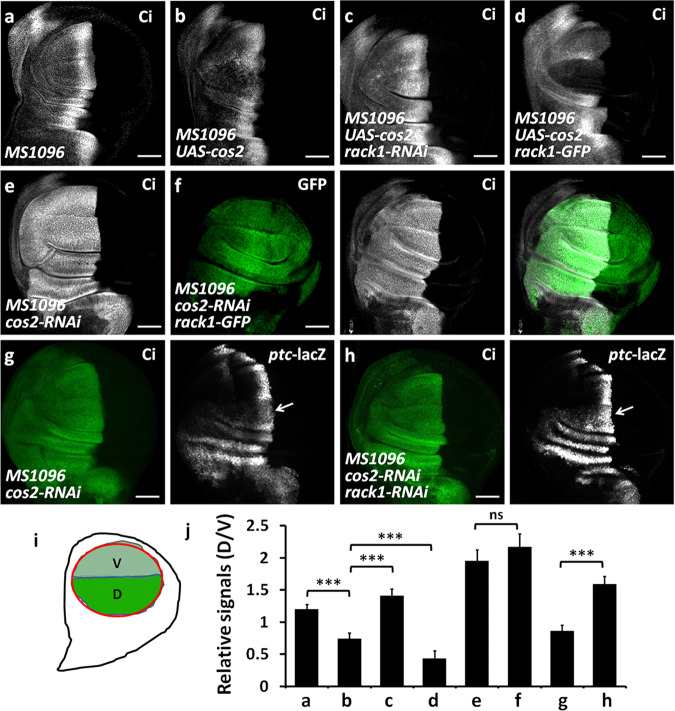
Rack1 synergizes with Cos2 to decrease Ci. **a**–**d** A control wing disc (**a**) and the discs expressing *cos2* alone (**b**) or *cos2* plus *rack1*-RNAi together (**c**) or *cos2* plus *rack1*-GFP simultaneously (**d**) by *MS1096* were stained to show Ci (white). Of note, the decrease of Ci induced by *cos2* overexpression was compromised by *rack1* knockdown, but aggravated by *rack1* overexpression. **e** A wing disc expressing *cos2* RNAi was stained with Ci antibody (white). Knockdown of *cos2* apparently increased anterior Ci. **f** A wing disc expressing *cos2* RNAi plus *rack1*-GFP was stained to show GFP (green) and Ci (white). Of note, knockdown of *cos2* rescued Ci decrease by *rack1* overexpression. **g**, **h** Wing discs expressing *cos2* RNAi alone (**g**) or *cos2* RNAi plus *rack1* RNAi together (**h**) were stained to show Ci (green) and *ptc*-lacZ (white). Notably, knockdown of *rack1* strengthened the increase of *ptc*-lacZ caused by *cos2* RNAi. **i** A schematic drawing showed the expression pattern of *MS1096*-Gal4. *MS1096*-Gal4 showed higher expression in the dorsal region (D) than ventral region (V) in the wing pouch. **j** Quantification analyses of **a**–**h** signals (*n* = 5). Scale bars: 50 μm for all wing discs.

In the cytoplasm, Ci should be phosphorylated by kinases prior to Slimb-mediated proteolysis [9, 10, 39]. However, Ci does not interact with these kinases, unless with assist of Cos2 [7]. Cos2 recruits kinases to Ci, leading to Ci phosphorylation on multiple sites [8]. Given that the above results showed that Rack1 regulated Slimb-mediated Ci degradation in the cytoplasm, we next examined the relationship between Rack1 and Cos2. Compared with the control disc (Fig. 3a), overexpression of *cos2* decreased Ci (Fig. 3b), which was alleviated by *rack1* RNAi (Fig. 3c) but aggravated by UAS-*rack1* (Fig. 3d). The previous studies have demonstrated that Hh signaling promotes Cos2 phosphorylation on S572 and S931 [17]. To test whether these phosphorylation modifications are involved in Rack1-related Ci regulation, we employed two mutant transgenic flies, Cos2-S572A and Cos2-927-935A (T927A, S931A, T934A, and S935A). Consistent with wild type Cos2, Rack1 promoted Cos2-S572A- and Cos2-927-935A-induced Ci decrease (Supplementary Fig. S4g–j), suggesting that these phosphorylation modifications are dispensable for Rack1 regulating Ci. Conversely, overexpression of *rack1* failed to decrease Ci under *cos2* RNAi background (Fig. 3e, f). In addition, knockdown of *cos2* led to *ptc*-lacZ upregulation in the anterior compartment cells (Fig. 3g), which was further boosted by simultaneous *rack1* knockdown (Fig. 3h). In sum, these data together suggest that Rack1 synergizes with Cos2 to downregulate Ci.

### Rack1 promotes Ci–Cos2 interaction

**Fig. 4:**
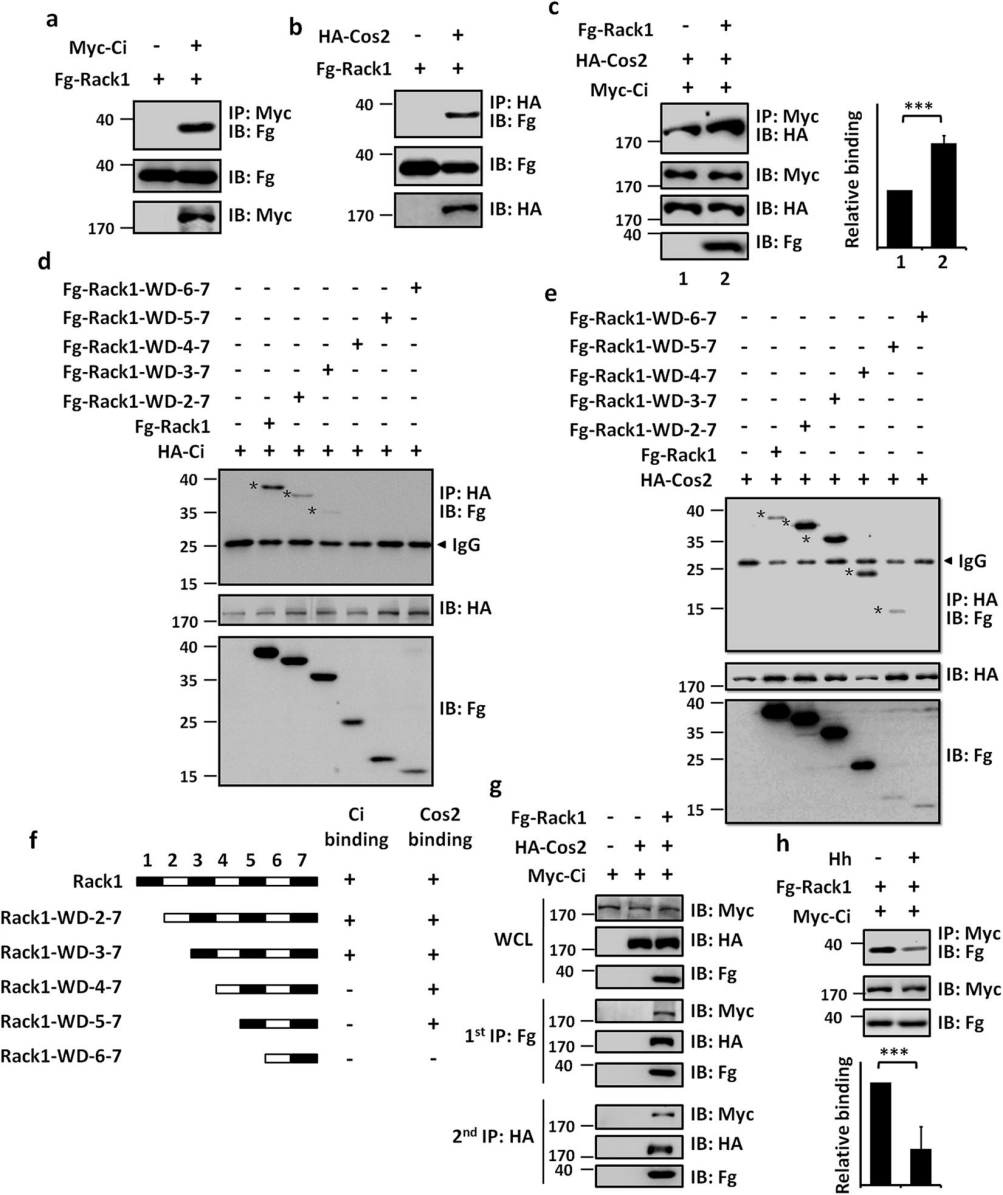
Rack1forms a ternary complex with Ci and Cos2. **a** Fg-Rack1 interacted with Myc-Ci in S2 cells. **b** Fg-Rack1 interacted with HA-Cos2 in S2 cells. **c** Fg-Rack1 enhanced the interaction between Ci and Cos2. Quantification analyses were shown on right (*n* = 3). **d** Immunoblots of immunoprecipitates (top) or lysates (bottom two panels) from S2 cells expressing indicated constructs. Asterisks indicate the binding strips. **e** The binding between Cos2 and distinct Rack1 truncated mutants. Asterisks indicate the binding strips. **f** Schematic drawings showed Rack1 truncates binding to Ci or Cos2. **g** S2 cells expressing the indicated proteins were harvested for the two-step immunoprecipitation and analyzed by western blot. Of note, Rack1, Cos2, and Ci formed a large complex. **h** Hh medium stimulation attenuated Myc-Ci binding to Fg-Rack1 in S2 cells. Quantification analyses were shown below (*n* = 3).

Since Rack1 protein only contains seven tandem WD40 domains, which always account for protein–protein interaction [26], it is necessary to test the binding between Rack1 and Ci. Through co-IP assays, we found that Ci could pull-down Rack1 (Fig. 4a). In addition, Cos2 also bound to Rack1 (Fig. 4b). Intriguingly, we revealed that co-transfection of Rack1 promoted Ci–Cos2 interaction (Fig. 4c).

To map which WD40 domain on Rack1 is responsible for Ci and Cos2 interactions, we generated various truncated mutants (Fig. 4f). The co-IP assay showed that constructs containing the third WD40 domain could bind Ci (Fig. 4d, f), indicating that the third WD40 domain possibly plays a role for its interaction with Ci. On the other hand, the fifth WD40 domain was important for Rack1–Cos2 interaction (Fig. 4e, f). Given that Rack1 could bind Ci and Cos2 through distinct WD40 domains, it is interesting to test whether these three proteins form a large complex. We performed a two-step immunoprecipitation (IP) experiment and found that Ci, Cos2, and Rack1 indeed formed a trimeric complex (Fig. 4g), suggesting that Rack1 may plays as a scaffold to bridge Ci and Cos2 together. We next sought to examine whether Hh signaling regulates Ci–Rack1 interaction, we carried out co-IP assays in S2 cells treated by a Hh-conditioned medium or control medium. Our data exhibited that Hh treatment robustly weakened the binding affinity between Ci and Rack1 (Fig. 4h).

### Rack1 promotes Slimb-mediated Ci processing

**Fig. 5:**
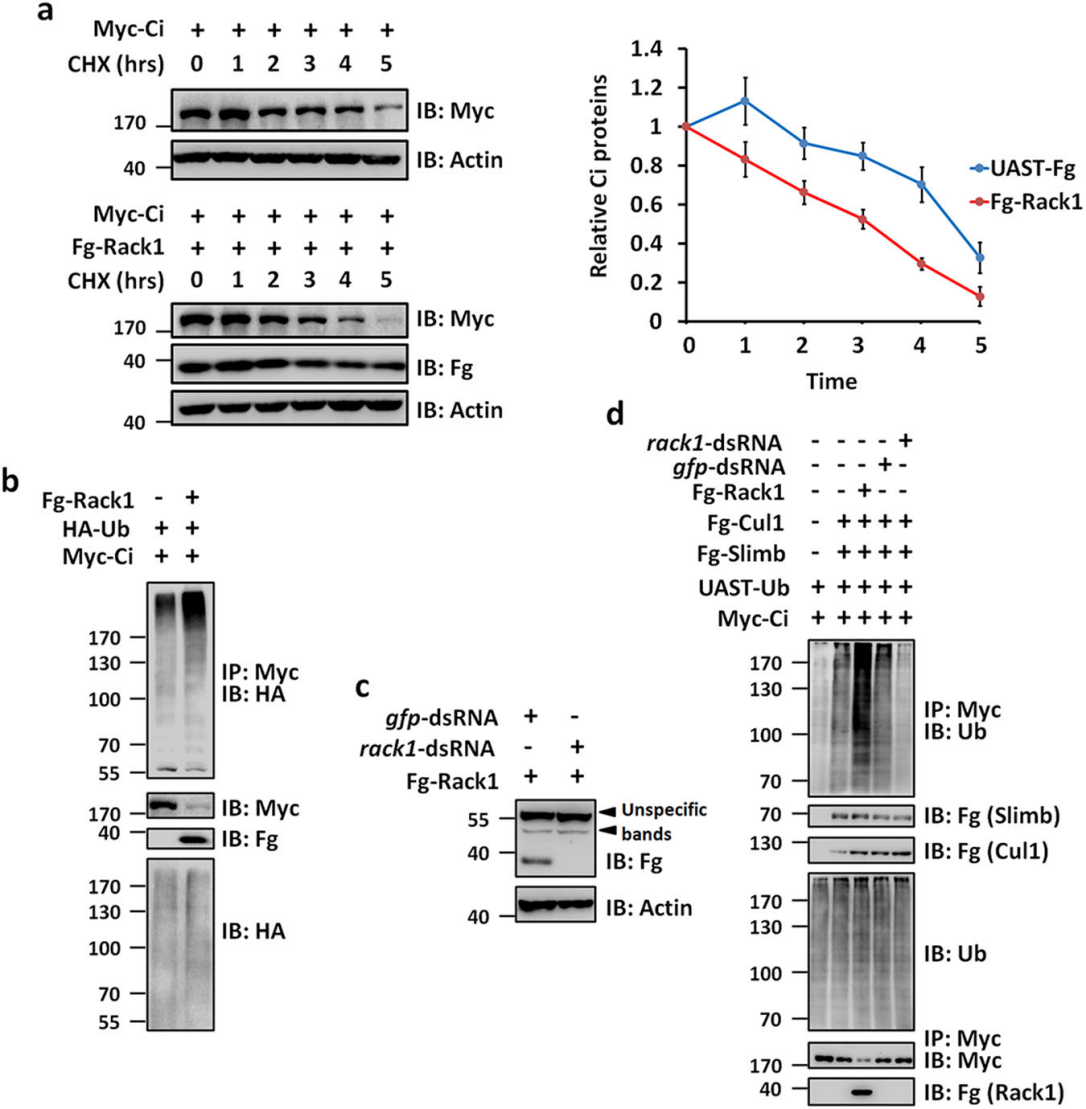
Rack1 promotes Slimb-mediated Ci degradation. **a** Immunoblots (IBs) of lysates from S2 cells expressing indicated proteins and treated with CHX for the indicated time intervals. Quantification analyses were shown on right of autoradiogram (*n* = 3). The results were presented as means ± SD of values from three independent experiments. Notably, Rack1 could promote Ci degradation. **b** IBs of immunoprecipitates (top) or lysates (bottom three panels) from S2 cells expressing indicated proteins and treated with MG132 for 4 h before cell harvesting. Overexpression of Rack1 elevated Ci ubiquitination. **c** *rack1*-dsRNA could silence the Fg-tagged Rack1 expression in S2 cells. **d** Overexpression of *rack1* increased, while *rack1* knockdown decreased Slimb-mediated Ci ubiquitination.

The above results have clearly demonstrated that Rack1 decreases Ci protein, but not affects *ci* transcription. Besides, Rack1 promotes Ci interaction with Cos2, implying that Rack1 may accelerates Slimb-mediated Ci degradation in the cytoplasm. Indeed, when transfected S2 cells were treated with translation inhibitor cycloheximide (CHX), Rack1 quickened the degradation of Ci (Fig. 5a). Since the ubiquitin modification is essential for Ci processing in the cytoplasm, we should investigate the ubiquitination status of Ci protein with or without Rack1. Through cell-based ubiquitination experiments, we found that co-transfection of Rack1 increased Ci ubiquitination (Fig. 5b). In contrast, we used dsRNA to silence the endogenous *rack1* and first confirmed that this dsRNA could effectively knockdown *rack1* (Fig. 5c). Overexpression of *rack1* promoted Slimb-Cul1-mediated Ci ubiquitination, while knockdown of *rack1* exerted an opposite effect (Fig. 5d). Overall, these results demonstrate that Rack1 decreases Ci protein through promoting Slimb-Cul1-mediated Ci ubiquitination.

### Rack1 enhances Smo cell surface expression

**Fig. 6:**
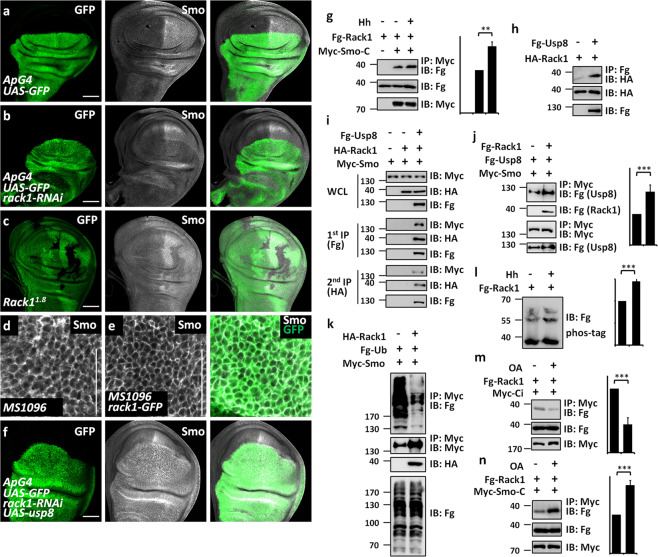
Rack1 stabilizes Smo through recruiting Usp8. **a**, **b** Wing discs of control (**a**) or expressing *rack1* RNAi by *ApG4* (**b**) was stained to show GFP (green) and Smo (white). Of note, knockdown of *rack1* decreased posterior Smo, but without apparent effect on anterior Smo. **c** A wing disc carrying *rack1*^*1.8*^ mutant clones was immunostained to show the expression of GFP (green) and Smo (white). Mutant clones are marked by the lack of GFP. Of note, *rack1* mutant clones exhibited decrease of Smo. **d** A control disc was stained to show endogenous Smo (white) in the posterior compartment region. **e** A wing disc expressing *rack1*-GFP was stained to show GFP (green) and Smo (white) in the posterior compartment region. Notably, overexpression of *rack1* elevated cell surface expression of Smo. **f** A wing disc expressing *rack1* RNAi plus *usp8* was stained to show GFP (green) and Smo (white). Overexpression of *usp8* restored Smo decrease by *rack1* knockdown. **g** Rack1 interacted with C-terminus of Smo in S2 cells, which was enhanced by Hh stimulation. Quantification analyses were shown on right (*n* = 3). **h** Fg-Usp8 bound HA-Rack1 in S2 cells. **i** S2 cells expressing the indicated proteins were harvested for the two-step immunoprecipitation and analyzed by western blot. Of note, Rack1, Smo and Usp8 formed a large complex. **j** Rack1 promoted the interaction between Smo and Usp8. Quantification analyses were shown on right (*n* = 3). **k** IBs of immunoprecipitates (top) or lysates (bottom three panels) from S2 cells expressing indicated proteins and treated with MG132 for 4 h before cell harvesting. Overexpression of Rack1 decreased Smo ubiquitination. **l** Hh treatment promoted Rack1 phosphorylation. Quantification analyses were shown on right (*n* = 3). **m**, **n** OA treatment weakened Ci–Rack1 interaction (**m**), while increased Smo–Rack1 interaction (**n**). Quantification analyses were shown on right (*n* = 3). Scale bars: 50 μm for all wing discs (**a**–**f**).

The previous study has demonstrated that Rack1 activates sonic Hh pathway through upregulating Smo abundance and cell membrane localization in non-small-cell lung cancer (NSCLC) cell lines [40]. In normal lung cells, Smo mainly localizes in the cytoplasm [40]. In NSCLC cells, the increased Hh ligands promotes Rack1 binding to Smo, resulting in Smo cell surface localization [40]. There are at least two questions needed to be addressed in this study. First, whether Rack1 is involved in regulating Smo abundance and localization in vivo under physiological condition? This study only pinpoints that Rack1 enhances Smo cell membrane accumulation under Hh stimulation in NSCLC cell lines. Second, how does Rack1 promote Smo cell surface localization? To address these questions, we employed wing disc as a tool. Compared with the control wing disc (Fig. 6a), knockdown of *rack1* decreased Smo (Fig. 6b). Furthermore, we confirmed this result using *rack* mutant clones (Fig. 6c). We also observed that loss of *rack1* only decreased Smo in posterior compartment cells, where *hh* expressed. This observation indicates that Rack1 possibly stabilizes Smo in the presence of Hh ligands. On the other hand, we found that overexpression of *rack1* indeed promoted Smo cell membrane localization (Fig. 6d, e), suggesting that Rack1 promotes Smo cell membrane localization under physiological status.

In the absence of Hh, the cytoplasmic Smo protein undergoes ubiquitin modification, leading to lysosome-mediated proteolysis [20, 21, 41]. With Hh stimulation, the ubiquitin chains are removed by deubiquitinases, culminating in Smo stabilization and cell membrane accumulation [19, 23, 24]. Since the ubiquitin modification plays an important role in regulating Smo localization, it is fruitful to test whether Rack1 promotes Smo stabilization and cell surface accumulation through Smo deubiquitination. We revealed that overexpression of *usp8* blocked *rack1* RNAi-induced Smo degradation (Fig. 6f), suggesting that Rack1 positively regulates Smo possibly through Usp8. The biochemical results revealed that Smo-C (C-terminus) interacted with Rack1, which was strengthened by Hh stimulation (Fig. 6g). In addition, Usp8 also pulled down Rack1 in S2 cells (Fig. 6h). The two-step co-IP assay showed that Usp8, Rack1, and Smo formed a trimeric complex (Fig. 6i). We further figured out that Rack1 could enhance the affinity between Usp8 and Smo (Fig. 6j), suggesting that Rack1 may acts as a scaffold to pull Usp8 and Smo together. The cell-based ubiquitination assay revealed that Rack1 indeed attenuated Smo ubiquitination (Fig. 6k). Overall, these results together imply that Rack1 stabilizes Smo and increases Smo cell membrane localization through promoting Smo–Usp8 interaction.

We next sought to test how does Hh signal modulate Ci–Rack1 and Smo–Rack1 interactions. The previous study had shown that the phosphorylation of Rack1 by AMPK regulated its affinity to autophagy complex [30], suggesting that the phosphorylation status of Rack1 affects its association with partners. Thus, we tested whether Hh signal regulates Rack1 phosphorylation. Strikingly, Hh conditional medium treatment induced a mobility shift of Fg-Rack1 on a phos-tag gel that reduces the mobility of phosphorylated proteins (Fig. 6l). Furthermore, when transfected S2 cells were treated with okadaic acid (OA, a phosphatase inhibitor) before cell harvesting, Rack1 showed a decreased binding to Ci (Fig. 6m), while elevated binding to Smo (Fig. 6n).

### Mammalian Rack1 plays a conserved role in regulating Ci and Smo

**Fig. 7:**
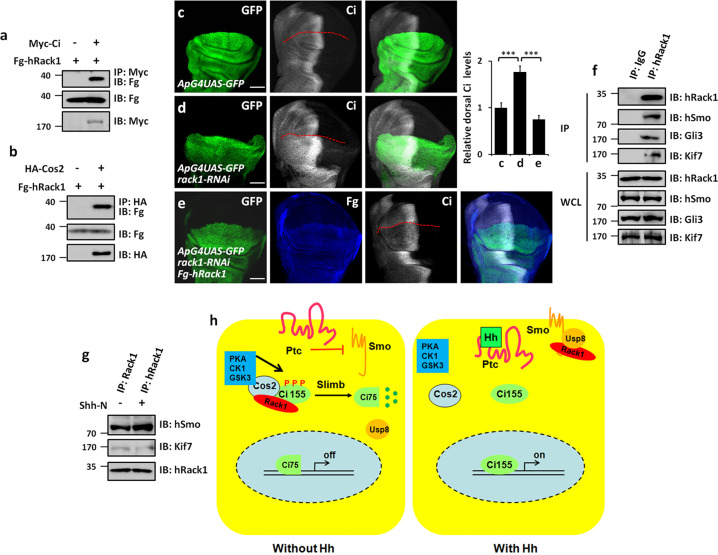
The regulation of Rack1 on Hh pathway is conserved in mammalian cells. **a** hRack1 interacted with Ci in S2 cells. **b** hRack1 bound Cos2 in S2 cells. **c**–**e** Wing discs of control (**c**), *rack1* RNAi (**d**), and simultaneously expressing Fg-tagged *hRack1* and *rack1* RNAi (**e**) was stained to show GFP (green), Fg (blue), and Ci (white). Quantification analyses were shown on right (*n* = 5). Of note, overexpression of *hRack1* could rescue Ci upregulation induced by *rack1* knockdown. **f** IBs of immunoprecipitates (top four panels) or lysates (bottom four panels) from HEK 293T cells. Notably, hRack1 could pull-down hSmo, Gli3, and Kif7. **g** Shh-N stimulation strengthened hRack1–hSmo interaction, while diminished hRack1–Kif7 association. **h** A proposed model of Rack1 regulating Hh pathway. In the absence of Hh, Rack1 forms a complex with Ci and Cos2, leading to Slimb-mediated Ci degradation (left). In the presence of Hh, Rack1 dissociates from Ci–Rack1–Cos2 complex and recruits Usp8 to Smo, resulting in Smo deubiquitination and cell surface accumulation (right). Scale bars: 50 μm for the wing disc (**c**–**e**).

The regulation of Hh pathway transduction is highly conserved from *Drosophila* to mammals [42]. Without Hh ligands, Sufu and Kif7 (the ortholog of Cos2) form a large complex with Gli2/3 and recruit the kinases including PKA, GSK3, and CK1 to phosphorylate Gli2/3 [7, 43]. The phosphorylated Gli2/3 undergoes the ubiquitination and degradation to generate C-terminal truncated repressors (Gli2-R and Gli3-R), which enter into the nucleus to inhibit the expression of Hh target genes [44]. With Hh ligands, Hh interacting with receptors unleashes Smo activity, leading to Smo enrichment on the cell membrane [45, 46]. Human hRack1 protein share 75.8% amino acid sequence identity with *Drosophila* Rack1, indicating there are evolutionarily conserved. Co-IP results showed that hRack1 could bind Ci and Cos2 in S2 cells (Fig. 7a, b). The increase of Ci protein induced by *rack1* RNAi could be restored by overexpressing hRack1 (Fig. 7c–e), suggesting that hRack1 can substitute *Drosophila* Rack1 to regulate Ci. Furthermore, we examined the protein–protein interactions using 293T cells and found that hRack1 could pull-down hSmo, Gli3, and Kif7 (Fig. 7f). In line with the finding in *Drosophila*, Shh-N treatment also decreased hRack1–Kif7 interaction, but increased hRack1–hSmo binding (Fig. 7g), indicating this regulatory mechanism is conserved in mammalian system.

## Discussion

Herein, we have discovered that the scaffold protein Rack1 plays dual roles in Hh signaling transduction. In the absence of Hh, Rack1 bridges Cos2 with Ci, leading to Slimb-mediated Ci processing in the cytoplasm (Fig. 7h). In the presence of Hh, Hh dissociates Ci–Rack1 interaction, and promotes Rack1 forming a trimeric complex with Usp8 and Smo, resulting in Smo deubiquitination and cell surface accumulation (Fig. 7h). Furthermore, we demonstrate that this regulation is conserved in mammalian system. Our findings thus unveil a fine-tuned mechanism by which Hh signal controlling Ci processing and Smo localization through Rack1 to achieve optimal pathway output.

Hh pathway exerts its function through the transcription factor Ci. Without Hh ligand, Ci forms a complex with Cos2 that recruits multiple kinases to phosphorylate Ci, promoting Slimb-mediated Ci proteolysis to generate a truncated form Ci-75 that blocks target gene expression. With Hh stimulation, Ci–Cos2–kinases complex dissociates via a yet unclear mechanism [8], resulting in full-length Ci accumulation. A lingering question is that how Hh stimulates the dissociation of Ci–Cos2–kinases complex. In this study, we show that Rack1 bridges Ci and Cos2 together to form a trimeric complex, which improves the interaction between Ci and Cos2. Hh decreases the affinity between Ci and Rack1, leading to dissociation of Ci–Rack1–Cos2 complex. Our results provide an explanation that Hh dissociates Ci–Cos2–kinases complex via the scaffold Rack1. Furthermore, we show that Hh promotes Rack1 phosphorylation and OA treatment decreases Ci–Rack1 interaction, indicating that phosphorylation of Rack1 possibly regulates its affinity with Ci. We also tried to identify which amino acid residual on Rack1 is responsible for Hh-induced phosphorylation. Unfortunately, after replaced all serine and threonine residues with alanines, the phosphorylation of Rack1 had no apparent decrease.

Another key component of Hh pathway is the GPCR-like protein Smo, which transduces the extracellular signal into cytoplasm. Cell surface accumulation of Smo is essential for Hh pathway activation. Increasing observations have showed that the localization of Smo is governed by endocytic trafficking. Smo is predominantly localized to the lysosomes in anterior compartment cells, where it is lack of Hh ligand, while is enriched on the cell membrane in posterior compartment cells where *hh* expresses [23]. The previous studies have also revealed that Hh stimulation diminishes Smo internalization [47], suggesting that Hh plays an important role in keeping Smo cell surface expression. The subcellular localization of Smo is tightly controlled by its ubiquitination. Ubiquitination is a reversible process by which the ubiquitin is attached to the lysine of proteins [48]. The mutant form of Smo, which all lysines are replaced by arginines, exclusively localizes on the cell membrane and fails to response to Hh stimulation [49]. Ubiquitination results in Smo cytoplasmic retention, while deubiquitination promotes Smo cell surface accumulation [19, 21]. Although it is well documented that Hh stimulation prevents Smo ubiquitination, the underlying mechanism is still unclear. In this study, we provide evidence that loss of *rack1* only decreases posterior Smo, without obvious effect on anterior Smo, suggesting that Hh confers Rack1 modulating Smo. Through biochemical analyses, we find that Rack1 binds Smo and recruits the deubiquitinase Usp8, which is boosted by Hh stimulation. Thus, our results provide a mechanism by which Hh elevates Smo cell surface accumulation via promoting Smo–Rack1–Usp8 complex formation.

In this study, we have revealed that Rack1 plays dual roles in Hh pathway. In the absence of Hh, Rack1 promotes Ci interaction with Cos2, leading to Slimb-mediated Ci proteolysis. In the presence of Hh, Rack1 dissociates from Ci–Rack1–Cos2 complex and binds Smo to recruit Usp8, culminating in Smo deubiquitination and cell surface accumulation. Overall, Rack1 plays dual roles in regulating Hh pathway.

The previous study has shown that hRack1 is upregulated in NSCLC samples and promotes NSCLC tumorigenesis via activating hSmo [40]. From this perspective, hRack1 acts as an oncogene in NSCLC. However, our study demonstrates that hRack1 could also function as tumor suppressor through promoting the degradation of Gli proteins. The hyperactivation of Hh pathway is thought to be one of the most important mechanisms driving NSCLC tumorigenesis. Most NSCLC cells show upregulation of Hh ligand [50]. In this case, the interactions between hRack1 and Gli proteins are inhibited, whereas hRack1–hSmo complex is strengthened, conferring hRack1 as an oncogene. On the other hand, some kinds of Hh-related cancers attribute to overexpression of Gli proteins, including breast cancer and prostate cancer [50]. In these cancers, hRack1 likely plays an antitumor role via binding Gli proteins. It is easy to accept that constitutive activation of Hh signaling in different types of human cancers adopt distinct mechanisms. Overall, the previous finding, together with our observations herein, raise a noteworthy concern that we should be scrupulous to choose hRack1 as therapeutic target for Hh-related cancers.

## Methods and materials

### DNA constructs

To generate Myc-Ci, Myc-Smo, HA-Rack1, HA-Ci, HA-Ub, Fg-Rack1, Fg-Cul1, Fg-Slimb, Fg-Usp8, Fg-hRack1, and Fg-Ub constructs, we amplified the corresponding cDNA fragments using Vazyme DNA polymerase (P505), and cloned them into the pUAST-Myc, pUAST-HA or pUAST-Fg backbone vectors respectively. UAS-Rack1-Y229/247F was generated via PCR-based site-directed mutagenesis. Truncated constructs including Rack1-WD2-7, Rack1-WD3-7, Rack1-WD4-7, Rack1-WD5-7, and Smo-C were generated by inserting the corresponding coding sequences into the indicated backbone vectors. A nuclear localization signal (NLS) from SV40 (PPKKKRKV) was inserted at the C terminus to generate Rack1-NLS plasmid. A myristoylation signal from Src (MGSSKSKPKDPSQRRRSLE) was inserted at the N terminus to generate Myr-Rack1. The GFP coding sequence was fused to the N terminus of Rack1 to get Rack1-GFP.

### Fly stocks and transgenes

Some stocks used in this study are kindly from Dr. Qing Zhang lab, such as *MS1096* (BDSC #8860), *ApG4* (BDSC #3041), *dpp*-lacZ [51], *kn*-lacZ [16], *ptc*-lacZ (BDSC #10514), *En-gal4* (BDSC #6356), HA-*rdx* [16], and Fg-*slimb* [16]. UAS-*usp8* was described previously [52]. UAS-GFP (BDSC #1522), *nub*-gal4 (BDSC #42699), *rack1*-RNAi (VDRC #104470, BDSC #34694), *rack1*^*1.8*^ (BDSC #24152), *mirr*-G4 (BDSC #29650), *ci*-lacZ (BDSC #6303), GMR-gal4 (BDSC #8605), *cos2*-RNAi (BDSC #44472), UAS-*cos2* (BDSC #67187), UAS-*cos2*-S572A (BDSC #55046), UAS-*cos2*-927-935A (BDSC #55047), *PKC53E*-RNAi (NIG #6622R-1), *PKC98E*-RNAi (NIG #GL00174), *PKN*-RNAi (NIG #2055R-1), *PKCδ*-RNAi (BDSC #28355), *aPKC*-RNAi, UAS-*PKCi* (BDSC #4589), *Src42A*-RNAi (NIG #HMS02755), *Src64B*-RNAi (NIG #HMC03327), and *slimb*-RNAi (NIG #3412R-1, 3412R-3) were obtained from BDSC or NIG. The detailed information of *ptc*-lacZ, *dpp*-lacZ, *kn*-lacZ, and *ci*-lacZ reporters have been described in FlyBase database (available at http://flybase.bio.indiana.edu/). UAS-*rack1*, UAS-*rack*1-GFP, UAS-*rack1*-NLS, UAS-Myr-*rack1*, UAS-Fg-*hRack1*, and UAS-*rack1*-Y229/247F transgenic flies were generated by injection of indicated constructs into *Drosophila* embryo according to the method described previously [16]. All stocks used in this study were maintained and raised under standard conditions. For all fly crosses, six virgins mated with four young males in a vial at 25 °C.

### Generating clones

Clones of mutant cells were generated by FLP/FRT-mediated mitotic recombination as previously described [34]. Genotypes of the *rack1* mutant clones were as follows: *hs-flp*; *rack1*^*1.8*^ FRT40A/*ubi*-GFP FRT40A. For mutant clone generation, the 1st larvae were heat shock at 37 °C for 1.5 h and kept at 25 °C for 2–3 days. The 3rd larvae were subjected to dissection according to standard protocols. Loss of GFP expression marked the mutant clones.

### Immunostaining and confocal

Immunostaining of discs were performed with previous protocols [53]. In brief, third-instar larvae were dissected in PBS and fixed in freshly made 4% formaldehyde in PBS at room temperature for 20 min, then washed three times with PBT (PBS plus 0.1% Triton X-100). Larvae were incubated overnight with needed primary antibodies in PBT at 4 °C, then washed with PBT for three times and incubated with corresponding fluorophore-conjugated secondary antibody 2 h at room temperature. After washed for three times in PBT, discs were dissected and mounted in 40% glycerol. Images were captured with Zeiss confocal microscope. Antibodies used in this study were as follows: rat anti-Ci (1:50; 2A1, DSHB); mouse anti-β Gal (1:500; 40-1a, Santa Cruz); mouse anti-Fg (1:1000; M2, Sigma); mouse anti-Lamin C (1:50; LC28.26, DSHB); mouse anti-HA (1:200; F-7, Santa Cruz); mouse anti-Smo (1:50; 20C6, DSHB); mouse anti-Ptc (1:50; Apa1, DSHB) and DAPI (1:1000; sc-3598, Santa Cruz). Secondary antibodies used in this study were bought from Jackson ImmunoResearch, and were diluted at 1:500. For immunostaining, at least 30 indicated discs were dissected for staining. The quantitative analysis was carried out using five random discs with correct genotypes.

### Cell Culture, transfection, immunoprecipitation, and immunoblotting

S2 cells (gift from Dr. Erjun Ling lab) were maintained in Schneider’s *Drosophila* Medium (Gibco) with 10% heat-inactivated FBS (Gibco) and 1% penicillin/streptomycin (Sangon Biotech). 293T cells were purchased from the ATCC and cultured in Dulbecco’s modified Eagle’s medium (Gibco) containing 10% FBS and 1% penicillin/streptomycin. Before experiments, cells were tested to be out of mycoplasma contamination. Cells were transfected using PEI (Sigma) according to the manufacturer’s instructions. Forty-eight hours after transfection, cells were harvested for IP and immunoblotting (IB) analysis with standard protocols as described [24]. Two-step IP experiments mentioned in this study were performed according to the protocol described previously [16]. To knockdown *rack1* in S2 cells, the double stranded RNA (dsRNA) was generated by MEGAscript High Yield Transcription Kit (Ambion) according to the manufacturer’s instructions. Rack1 dsRNA targets the full-length coding sequence. dsRNA targeting the *gfp* full-length coding sequence was used as a negative control. The dsRNA was transfected into S2 cells by ExFect 2000 (Vazyme) according to the manufacturer’s instructions. For OA treatment, transfected S2 cells were treated with 10 μM OA (CST) for 2 h before cell harvesting. 10% acrylamide phos-tag gel with 20 μM phos-tag (Wako) acrylamide was used to examine Rack1 phosphorylation according to the manufacturer’s instructions. The following antibodies were used for IB: mouse anti-HA (1:2000; F-7, Santa Cruz); mouse anti-Myc (1:2000; 9E10, Santa Cruz); mouse anti-Fg (1:5000; M2, Sigma); mouse anti-Ub (1:1000; P4D1, Santa Cruz); mouse anti-Actin (1:5000; A00702, Genscript); mouse anti-hRack1 (1:1000; B-3, Santa Cruz); rabbit anti-hSmo (1:1000; A3274, ABclonal); rabbit anti-Gli3 (1:1000; A15613, ABclonal); rabbit anti-Kif7 (1:1000; A15581, ABclonal); goat anti-mouse HRP (1:10,000; ABCA2512079, Abmax) and goat anti-rabbit HRP (1:10,000; ABCA2511749, Abmax).

### Protein stability assay and ubiquitination assay

Protein pulse-chase experiments were performed as previously described [16]. Briefly, S2 cells were plated in 10 cm dishes and transfected with indicated plasmids. After 24 h, the cells were transferred into 12-well cell culture plates at equivalent densities. Cells were treated with 20 μg/ml CHX (Calbiochem) for the indicated intervals before harvesting. IB experiments were carried out to examine the levels of indicated proteins. For cell-based ubiquitination assays, S2 cells were transiently transfected with the indicated combinations of vectors. Four hours before cells harvesting, MG132 were added to the media to block proteasome-mediated proteolysis. The ubiquitination assays were then carried out according to the previously described protocol [16]. In brief, cells were lysed with denaturing buffer (1% SDS, 50 mM Tris-base, pH 7.5, 0.5 mM EDTA, and 1 mM DTT) and incubated at 100°C for 5 min. The lysates were then diluted tenfold with regular lysis buffer and subject to IP and western blot analysis.

### RNA isolation, reverse transcription, and real-time PCR

About 40 wing discs were lysed in TRIzol (Invitrogen) for RNA isolation according to previous described [54]. One microgram RNA was used for reverse transcription by HiScript^®^ Q RT SuperMix with gDNA wiper (Vazyme) according to the instructions. Real-time PCR was performed on BIO-RAD CFX96^TM^ with ChamQ SYBR^®^ Color qPCR Master Mix (Q411, Vazyme). 2^−∆∆Ct^ method was used for relative quantification. The primer pairs used were as follows: *rack1*, 5′-GAGCACAACGACATCATC-3′ (forward) and 5′-CTCC-TCAACGGTCTTCTT-3′ (reverse); *ci*, 5′-AGCGAGTAATACTTCGGTTA-3′ (forward) and 5′-TGATTGGTAGATGTGTTGTTC-3′ (reverse); *ptc*, 5′-AGATCGG- GCAAATCCTATG-3′ (forward) and 5′-GTCAGCGATGGATCATTAAG-3′ (reverse); *dpp*, 5′-CCTGGTCAACAATATGAATCC-3′ (forward) and 5′-TCATCTCCTGGTA-GTTCTTC-3′ (reverse); *kn*, 5′-CTTCAATTCCACGTCAAGG-3′ (forward) and 5′-TATAGCCCTGTGTATTGCA-3′ (reverse); *actin*, 5′-GTACCCCATTGAGCACGG-TA-3′ (forward) and 5′-CGAACATGATCTGGGTCATC-3′ (reverse). Data are presented as means ± SD of values from three experiments.

### Statistical analysis

The density of western blot band or immunostaining was measured by Image J software. Statistical analysis was performed with GraphPad Prism software. The data shown in the Figures were representative of three or more independent experiments and were analyzed by one way student’s *t* test, and *P* < 0.05 was considered statistically significant. Where exact *P* values are not shown, statistical significance is shown as with ns, no significance, *P < 0.05, **P < 0.01 and ***P < 0.001.

## Supplementary information

FigureS1

FigureS2

FigureS3

FigureS4

Supplementary information
