# Supplementary figures and images for: Dual functions of Rack1 in regulating Hedgehog pathway
Source: Cell Death Differ. 2020 May 28;27(11):3082–96. doi: 10.1038/s41418-020-0563-7 (PMC7560836; doi:10.1038/s41418-020-0563-7)

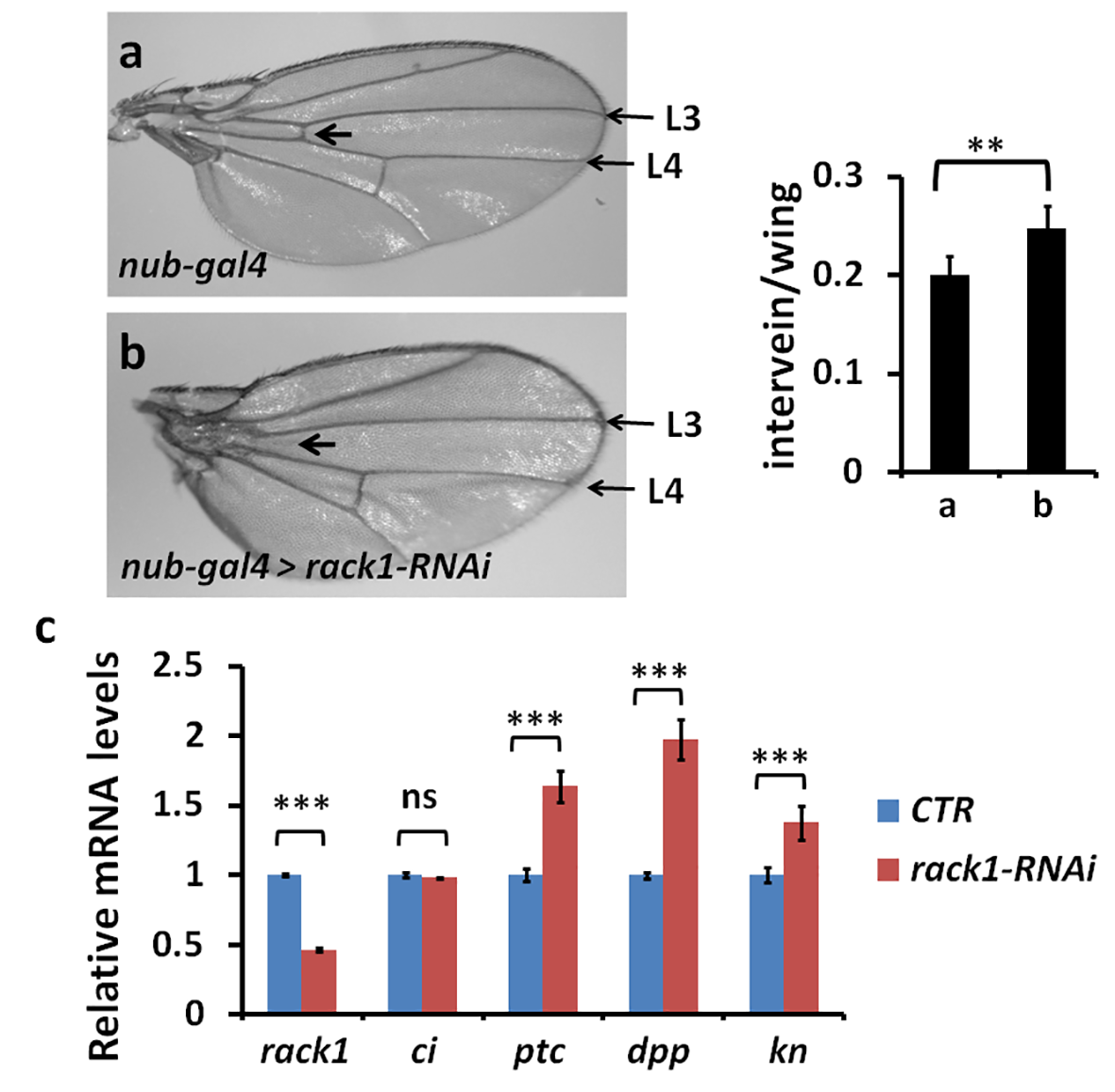

Supplement: Supplementary file 1 — FigureS1 [file 41418_2020_563_MOESM1_ESM.tif]

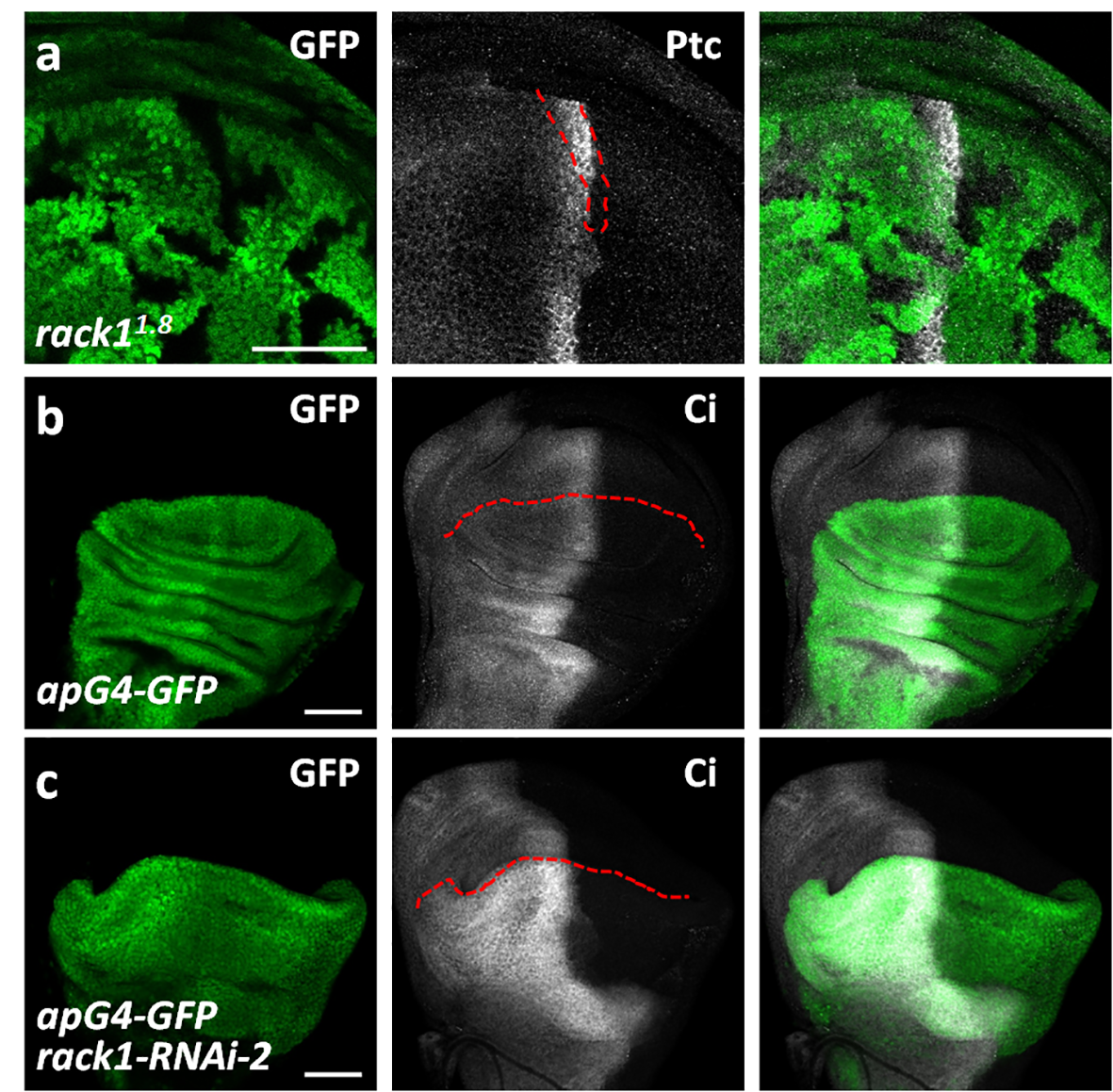

Supplement: Supplementary file 2 — FigureS2 [file 41418_2020_563_MOESM2_ESM.tif]

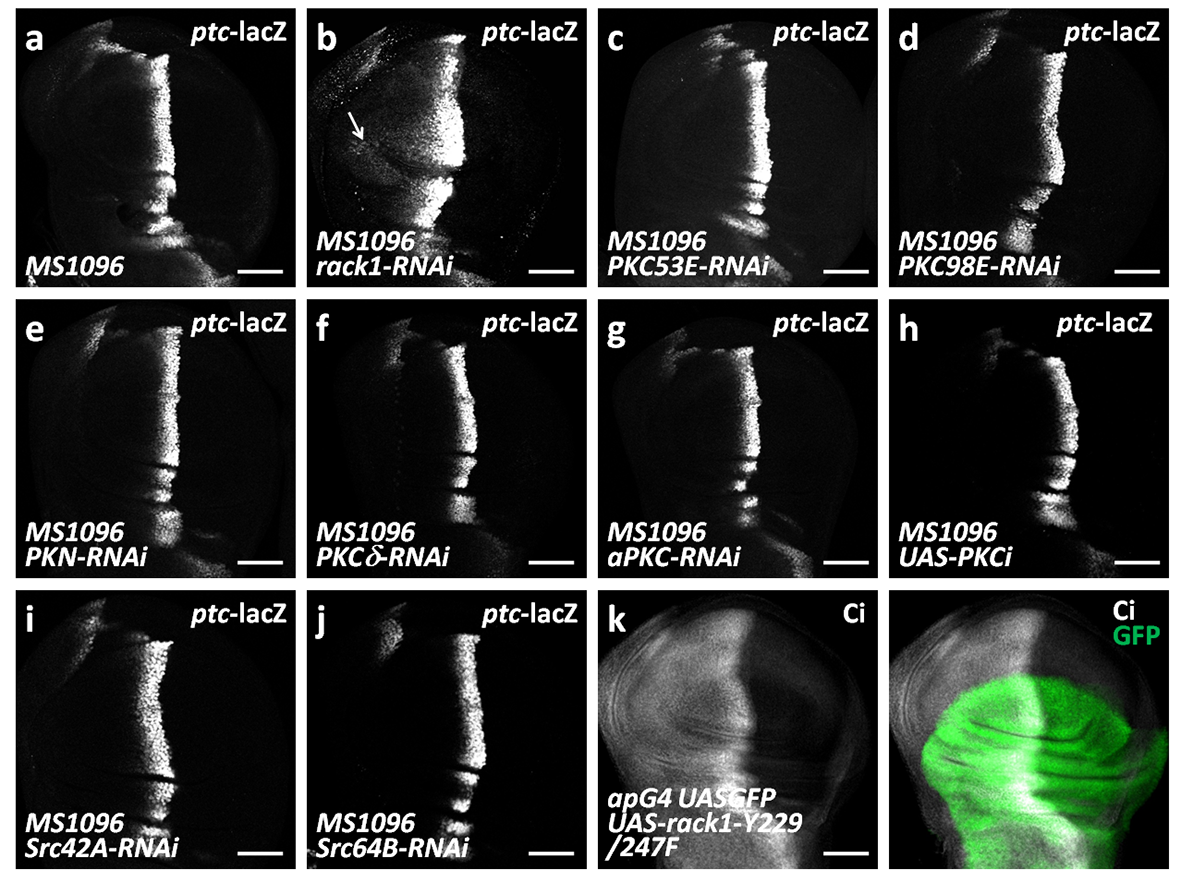

Supplement: Supplementary file 3 — FigureS3 [file 41418_2020_563_MOESM3_ESM.tif]

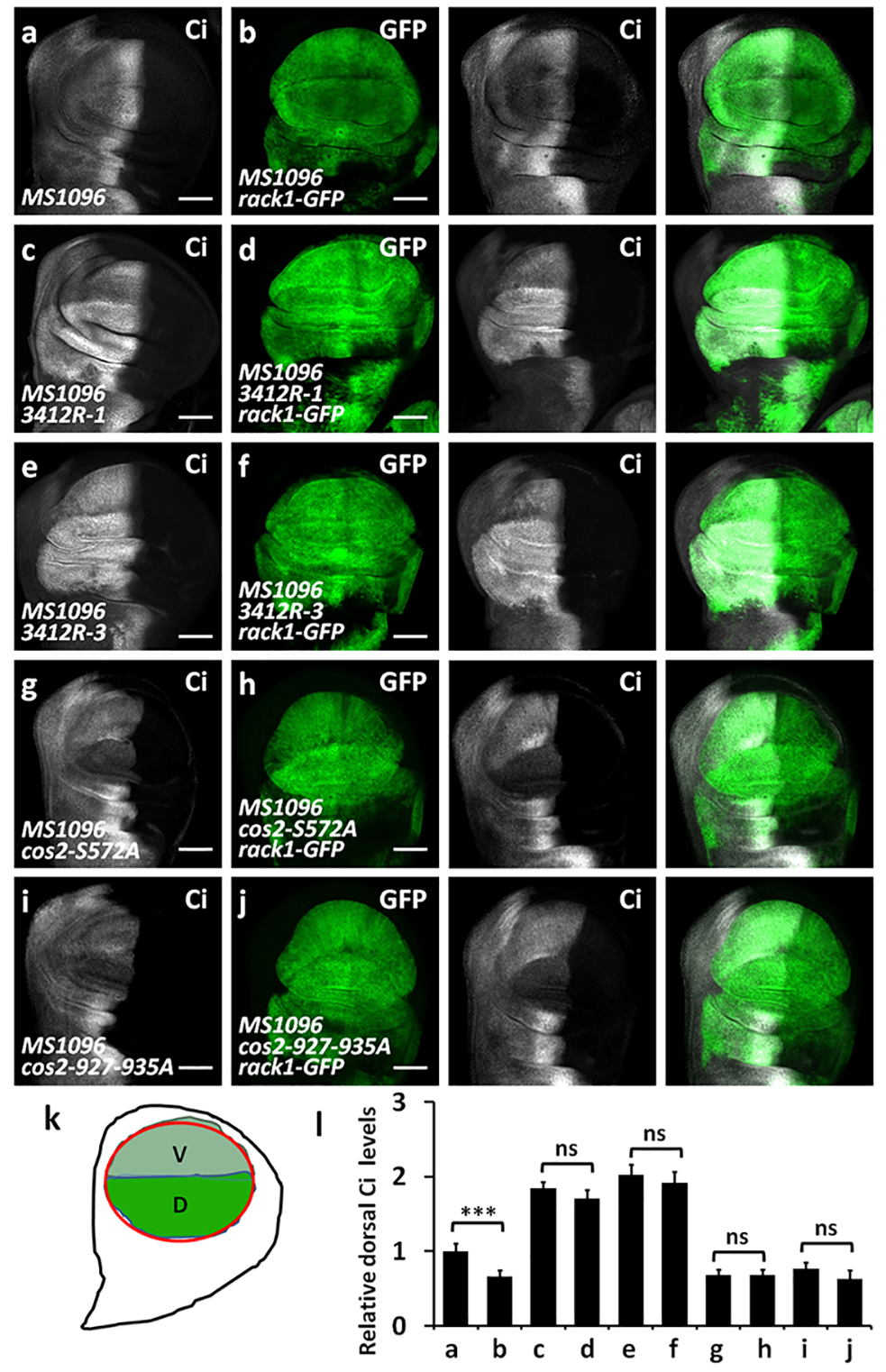

Supplement: Supplementary file 4 — FigureS4 [file 41418_2020_563_MOESM4_ESM.tif]
