# Supplementary material for: Dual functions of Rack1 in regulating Hedgehog pathway
Source: Cell Death Differ. 2020 May 28;27(11):3082–96. doi: 10.1038/s41418-020-0563-7 (PMC7560836; doi:10.1038/s41418-020-0563-7)
Supplement: Supplementary file 5 — Supplementary information [file 41418_2020_563_MOESM5_ESM.docx]

**Supplementary Information**

Dual functions of Rack1 in regulating Hedgehog pathway

Li et.al

**Supplementary Figure 1. Knockdown of *rack1* widened the space between vein3 and vein4. a** A control wing. **b** A wing expressing *rack1* RNAi by *nub*-gal4. Quantification analyses were shown on the right. The results were presented as means±SD of values from ten wings. Of note, knockdown of *rack1* increased the width between vein3 and vein4. **c** Real time PCR of *nub*-gal4 (CTR) or *nub*-gal4> *rack1*-RNAi wing discs. Knockdown of *rack1* elevated Hh target genes expression. Above all, ns, no significance, **P﹤0.01 and ***P﹤0.001.

**Supplementary Figure 2. Loss of *rack1* activated Hh pathway. a** A wing disc carrying *rack1^1.8^* mutant clones was immunostained to show the expression of GFP (green) and Ptc (white). Mutant clones are marked by the lack of GFP. Of note, *rack1* mutant clones exhibited elevation of Ptc. **b-c** A control wing disc (**b**) and a wing disc expressing *rack1* RNAi by *ApG4* (**c**) was stained to show GFP (green) and Ci (white). Knockdown of *rack1* increased Ci. Scale bars: 50μm for all wing discs. Above all, ns, no significance and ***P﹤0.001.

**Supplementary Figure 3. Rack1 regulates Hh pathway independent of PKC and Src.** **a** A control wing disc was stained to show *ptc*-lacZ (white). **b** A wing disc expressing *rack1* RNAi by *MS1096* was stained to show *ptc*-lacZ (white). Knockdown of *rack1* increased *ptc*-lacZ. **c**-**g** Wing discs expressing *PKC53E* RNAi (**c**), *PKC98E* RNAi (**d**), *PKN* RNAi (**e**), *PKCδ* RNAi (**f**), and *aPKC* RNAi (**g**) were stained to show *ptc*-lacZ (white). Knockdown of any PKC kinases did not affect *ptc*-lacZ expression. **h** A wing disc expressing *PKCi* by *MS1096* was stained to show *ptc*-lacZ (white). **i, j** Wing discs expressing *Src42A* RNAi (**i**) and *Src64B* RNAi (**j**) were stained to show *ptc*-lacZ (white). Of note, knockdown of any Src kinases did not affect *ptc*-lacZ expression. **k** A wing disc expressing *rack1*-Y229/247F by *ApG4* was stained to show Ci (white) and GFP (green). Scale bars: 50μm for all wing discs.

**Supplementary Figure 4. Rack1 decreases Ci through Slimb. a-b** A wing disc of control (**a**) and expressing *rack1*-GFP by *MS1096* (**b**) were stained to show GFP (green) and Ci (white). **c, e** Wing discs expressing *slimb* RNAi (3412R-1 or 3412R-3) by *MS1096* were stained to show Ci (white). Knockdown of *slimb* apparently increased Ci. **d, f** Wing discs simultaneously expressing *slimb* RNAi and *rack1*-GFP were immunostained to show GFP (green) and Ci (white). Overexpression of *rack1* did not decrease Ci in *slimb* RNAi background. **g** A wing disc expressing *cos2*-S572A was stained to show Ci (white). **h** A wing disc expressing *cos2*-S572A plus *rack1*-GFP was stained to show GFP (green) and Ci (white). Rack1 aggravated Cos2-S572A-mediated Ci decrease. **i** A wing disc expressing *cos2*-927-935A was stained to show Ci (white). **j** A wing disc expressing *cos2*-927-935A plus *rack1*-GFP was stained to show GFP (green) and Ci (white). Of note, Rack1 aggravated Cos2-927-935A-mediated Ci decrease. **k** A schematic drawing showed the expression pattern of *MS1096*-Gal4. *MS1096*-Gal4 showed higher expression in the dorsal region (D) than ventral region (V) in the wing pouch. **l** Quantification analyses of **a**-**j** Ci signals (*n*=5). Scale bars: 50μm for all wing discs. Scale bars: 50μm for all wing discs. Above all, ns, no significance, **P﹤0.01 and ***P﹤0.001.
